# Faecal avoidance and selective foraging: do wild mice have the luxury to avoid faeces?^☆^

**DOI:** 10.1016/j.anbehav.2013.06.011

**Published:** 2013-09

**Authors:** Patrick T. Walsh, Erin McCreless, Amy B. Pedersen

**Affiliations:** a Institute of Evolutionary Biology, School of Biological Sciences, University of Edinburgh, Edinburgh, U.K.; b Department of Ecology and Evolutionary Biology, University of California Santa Cruz, Santa Cruz, CA, U.S.A.; c Centre for Immunity, Infection & Evolution, School of Biological Sciences, University of Edinburgh, Edinburgh, U.K.

**Keywords:** antiparasite behaviour, faecal avoidance, faecal–oral transmission, feeding behaviour, laboratory mouse, parasite, *Peromyscus*, selective foraging, wild immunology

## Abstract

Host–parasite interactions are a key determinant of the population dynamics of wild animals, and behaviours that reduce parasite transmission and infection may be important for improving host fitness. While antiparasite behaviours have been demonstrated in laboratory animals and domesticated ungulates, whether these behaviours operate in the wild is poorly understood. Therefore, examining antiparasite behaviours in natural populations is crucial for understanding their ecological significance. In this study, we examined whether two wild rodents (white-footed mice, *Peromyscus leucopus*, and deer mice, *Peromyscus maniculatus*), selectively foraged away from conspecific faeces or avoided faeces altogether, and whether faecal gastrointestinal parasite status affected their behaviour. We also tested whether wild mice, when nesting, avoided using material that had previously been used by healthy or parasite-infected conspecifics. Our results, in contrast to laboratory mouse studies, suggest that wild mice do not demonstrate faecal avoidance, selective foraging or selective use of nesting material; they preferred being near faeces and did not differentiate between faeces from parasitized and uninfected conspecifics. Behavioural avoidance to reduce parasite infection may still represent an important strategy; however, mice in our study population appeared to favour the opportunity to feed and nest over the risks of coming into contact with faecal-transmitted parasites. Furthermore, the presence of conspecific faeces may actually provide a positive cue of a good foraging or nesting location. Ultimately, balancing the trade-off of performing antiparasite behaviours to reduce infection with missing an important feeding or nesting opportunity may be very different for animals in the wild facing complex and stochastic environments.

## Highlights

• Parasites can be important for the fitness and dynamics of wild animals.
• Transmission by faecal–oral contact is common for parasites of wild animals.
• We examine whether wild mice selectively avoid faeces to reduce infection risk.
• Surprisingly, wild mice did not avoid faeces during foraging, feeding or nesting.
• Wild mice favour the chance to feed and nest over the risk of parasite contact.

Parasites play a major role in regulating the dynamics of wild animal populations (Anderson & May 1979). Hosts are known to employ a variety of methods, both physiological and behavioural, to avoid or eliminate parasites (Hart 1990, 1992; Loehle 1995). Immune and other physiological responses to parasites, defined here as both macroparasites (helminths, fungi, ectoparasites) and microparasites (viruses, bacteria, protozoans), are relatively well understood, but comparatively fewer studies have focused on specific antiparasite behaviours that can protect the host from infection and the possible fitness consequences of parasitism (Ezenwa 2004; Daly & Johnson 2011; de Roode & Lefèvre 2012). For a particular behaviour to be considered as reducing parasite contact or the likelihood of infection, two criteria must be met: (1) the parasite should have a negative effect on the host's fitness; and (2) the behaviour in question should be shown to be effective in helping an animal to avoid, remove or mitigate parasite infection (Hart 1990).

Animals can exhibit behaviours that may reduce the spread of pathogens to themselves and fellow group members (Moore 2002). Some of these behaviours are employed after parasites are already present. Grooming, for example, serves to remove or reduce ectoparasites and has been documented extensively across mammals (Hart 1990; Cotgreave & Clayton 1994). Similarly, self-medication, in which a species selectively feeds on resources that have medicinal qualities to eliminate or reduce parasite infection levels, has been documented in primates, including ingesting compounds that may be useful against helminths (e.g. Wrangham & Nishida 1983), and recently demonstrated in the ovipositing choices of infected monarch butterflies, *Danaus plexippus* (Lefèvre et al. 2012). However, gastrointestinal parasites, commonly spread through faecal–oral transmission, may require that different behaviours be employed to reduce parasite contact and the probability of infection in the first place. It appears that animals that use behavioural strategies to avoid parasite transmission are probably responding to cues from the infected individuals, rather than the direct presence of transmissible parasite stages (Cooper et al. 2000; Kavaliers et al. 2005). One behaviour that may reduce exposure to faecal–orally transmitted parasites is selective foraging, or preferentially foraging away from faeces (Hart 1990).

Selective foraging has been demonstrated to reduce parasite loads in animals (Michel 1955), but behavioural observations are largely limited to domesticated livestock and wild ungulates (e.g. cattle, *Bos taurus*: Michel 1955; domestic sheep, *Ovis aries*: Crofton 1958; Hutchings et al. 1999, 2000, 2002; Cooper et al. 2000; horses, *Equus caballus*: Odberg & Francis-Smith 1976, 1977; reindeer, *Rangifer tarandus*: Moe et al. 1999; van der Wal et al. 2000; wild antelopes: Ezenwa 2004; chamois, *Rupicapra rupicapra*: Fankhauser et al. 2008; exceptions: primates: Freeland 1980; macropodids: Garnick et al. 2010). Therefore, it is unclear how widely this behavioural adaptation occurs in other animals.

Equally, communal or sequential nest use, roosting sites and burrows potentially provide another significant risk for infection by faecal–orally transmitted parasites. Therefore, the detection and avoidance of previously used or faecal-contaminated nest or sleeping sites and materials may minimize parasite transmission and has been extensively studied in bird nesting behaviour (see Mazgajski 2007). However, while some bird species do avoid used or infected nests (e.g. Brown & Brown 1986; Merilä & Allander 1995), others show no avoidance (e.g. Johnson 1996; Blem et al. 1999) or even a preference for previously used nests (e.g. Jackson & Tate 1974; Davis et al. 1994). Wild rodents also use nests communally and sequentially (Wolff & Hurlbutt 1982; Frank & Layne 1992) and have been found to remove old materials from their nests, in subsequent uses, as a method of removing ectoparasites (e.g. house mice, *Mus musculus*: Schmid-Holmes et al. 2001; Brants' whistling rats, *Parotomys brantsii*: Roper et al. 2002). The diversity of behavioural responses to minimize the potential for parasite contact and infection suggests that antiparasite behaviours may represent a cost to the host, and that the benefit of avoiding parasites must outweigh the cost of not performing the behaviour, consequently exposing the host to potential infection (Hart 1990).

Rodents communicate primarily through olfaction, and may use olfactory cues to detect parasitized individuals. In studies of laboratory mice, females consistently spend more time near the urine of healthy males and preferentially mate with healthy individuals over individuals infected with gastrointestinal parasites (Penn & Potts 1998; e.g. *Heligmosomoides polygyrus*: Ehman & Scott 2001; *Eimeria vermiformis*: Kavaliers & Colwell 1995; Kavaliers et al. 1997). Yet, although laboratory mice have been shown to be able to detect parasitized individuals (Kavaliers et al. 2005), it has not been demonstrated whether this occurs in wild individuals or extends to selective foraging or selectivity in nesting sites or burrows.

Wild populations of white-footed mice, *Peromyscus leucopus*, and deer mice, *Peromyscus maniculatus*, are ideal for studying faecal avoidance and selective feeding. First, they live in high-density communities and have a high prevalence of intestinal parasites (Pedersen 2005; Clotfelter et al. 2007). Over 10 species of faecal–orally transmitted gastrointestinal parasites have been found to infect both species of *Peromyscus* at our study site, including helminths and protozoans (Pedersen & Greives 2008). Second, the burrows of these mice contain significant amounts of both faeces and stored food and are used by several individuals over short periods of time (Wolff & Hurlbutt 1982; Wolff 1985a, b; Wolff & Durr 1986), making nest sites likely locations for parasite transmission. Finally, there is evidence that the gastrointestinal parasites that infect *Peromyscus* can have negative fitness consequences. For example, *Eimeria* spp. infection has been associated with lower mass and overwintering survival (Fuller & Blaustein 1996) and, in this study population, antihelminthic treatment can, in part, limit seasonal population crashes (Pedersen & Greives 2008). Thus, mice may benefit from faecal avoidance or selective feeding or nesting to avoid contact and infection with these parasites.

In this study we investigated whether wild *Peromyscus* demonstrate faecal avoidance or differentiate between gastrointestinal parasite-infected or uninfected faeces when feeding or in nesting situations. Understanding behavioural adaptations to parasites in wild animals will provide insights into host–parasite dynamics and parasite-driven regulation of animal behaviours and population dynamics.

## Methods

### Study Site

These experiments were conducted at the Mountain Lake Biological Station (MLBS) in Giles County, Virginia, U.S.A. (3722′21′N, 8031′20′W, elevation: 1160 m above sea level). The site consists of oak–maple forest that supports large, coexisting populations of *P. leucopus* and *P. maniculatus* (Wolff 1996; Clotfelter et al. 2007; Pedersen & Greives 2008).

### Trapping Methods

Six 0.5 ha grids were trapped for 3 consecutive days every 2 weeks during the summer of 2002. Each grid had 64 Sherman live folding traps (5 × 2 cm and 16.5 cm high, H.B. Sherman; Tallahassee, FL, U.S.A.), spaced 10 m apart. In addition, mice were trapped on a separate 2.25 ha grid, in a similar habitat, once a month. Traps were set at dusk with crimped oats, and checked the following morning. All captured individuals were permanently ear tagged (National Band & Tag, U.S.A.), and species and sex were recorded. All individuals included in experimental trials were adult mice.

### Faecal Analysis

For all faecal samples used in the faecal avoidance, selective foraging and nesting material use experiments, a subsample was used to determine infection status. Faecal samples were obtained from individuals, and faecal float analysis (saturated sodium chloride) was used to identify gastrointestinal parasite infection (Pritchard & Kruse 1982). Eggs from each sample, concentrated on a cover slip, were identified to parasite species by scanning five transects under a microscope (100× magnification, Pedersen 2005). Samples without gastrointestinal parasites were identified as ‘healthy’ and samples that contained one or more of the following gastrointestinal parasites were considered ‘parasitized’: nematodes including *Aspiculuris americana*, *Capillaria americana* and *Syphacia peromysci*, and two unidentified morphospecies; protozoans including *Eimeria delicata* and *Eimeria arizonensis*; and cestodes including *Hymenolepis diminuta* and *Hymenolepis citelli* (Pedersen 2005; Pedersen & Antonovics 2013). The life cycles of this group of gastrointestinal parasites varies significantly, from the pinworms (*A. americana* and *S. peromysci*), which can be directly infectious after defecation, to the coccidial protozoans (*Eimeria* spp.), which require 10–14 days of development in the soil. The cestodes (e.g. *Hymenolepis* spp.) even require an intermediary arthropod host before they are infectious to the next mouse. We used 1–3-day old faeces, which will not contain infectious stages of many of the parasites, to test whether wild mice can detect cues in the faeces of parasitized mice that lead them to avoid feeding or nesting.

### Experiment 1: Faecal Avoidance and Selective Feeding

A choice test was used to determine whether wild *Peromyscus* exhibited faecal avoidance or selective feeding, or differentiated between faeces from infected and uninfected mice. In this experiment 136 wild *Peromyscus* were caught and used (114 *P. leucopus* and 22 *P. maniculatus*).

**Figure 1:**
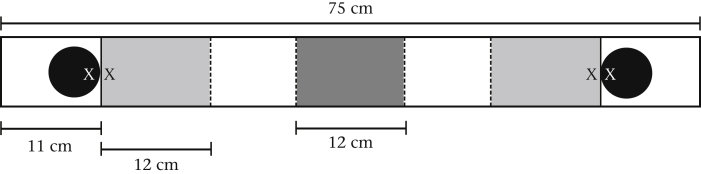
Diagram of the Plexiglas choice arena. The black circles indicate the position of the petri dishes, the white ‘X’ indicates the position of the faeces, the black ‘X’ the position of the food when present, and the solid lines the screens. The dashed lines correspond to actual lines drawn onto the bottom of the choice arena. The light-grey areas correspond to the two choice sections and the dark-grey area to the starting position.

All experimental trials were conducted between dawn and 1200 hours. After demographic data were collected on each mouse, individual mice were placed in the middle of a choice arena, which consisted of a rectangular Plexiglas box (75 × 10 cm and 7 cm high) with 11 × 10 cm areas blocked off by screens on each end (Fig. 1). Directly behind each screen was a petri dish in which either a healthy (containing no parasites) or parasitized (gastrointestinal parasite-contaminated) faecal sample could be placed. The control was an empty petri dish placed behind the screen. The healthy and parasitized faecal samples were each a mix of faeces from at least three different mice and included faeces from both sexes, to minimize the possibility that the focal mouse's behaviour would be driven by other olfactory cues. Five unique sets of faecal samples, which all contained eggs/oocysts from several parasite species, were used throughout the experiment to ensure that they were fresh when used in trials, and all samples were refrigerated between trapping days. All faecal samples contained the same weight of faecal material. The observer was blind to the status of the faecal samples, and the side of the choice arena where the samples or controls were placed was randomized. Between trials, the choice arena was cleaned with ethanol wipes and air-dried. For both faecal avoidance and selective foraging choice tests, three trials were run: (1) parasitized faeces versus healthy faeces; (2) parasitized faeces versus the control; and (3) healthy faeces versus the control.

At the start of each trial, a mouse was placed in a small permeable screen container in the centre of the choice arena for a 30 s adjustment period (Fig. 1). After the adjustment period, the container was removed and the mouse was allowed to explore the whole arena for 6 min. The arena was clearly divided, with lines across the base, into three 12 cm sections (Fig. 1). The central 12 cm section was marked off to ensure that the placement of the mouse was consistent across trials. The time a mouse spent in the 12 cm sections at either end of the box was recorded with a stopwatch. A mouse was determined to be in a section if its entire body, excluding its tail, had crossed the line into that section. The mouse was deemed to be in the centre when it was not in either of the two opposite end sections. At the end of the choice trial the mouse was released at the same trap location where it had been captured. Each mouse was used for a single experimental trial before it was released, so that no mouse was used for more than one test on the day it was captured. Of the 136 mice, 18 (13.24%) were used for more than one test, but these were conducted on different days. Excluding these individuals does not change the results, so the stated results include these individuals.

The same experimental design was used to test both faecal avoidance and selective foraging. Faecal avoidance was examined, as described above, by exposing the mouse to faeces or control in the three trials (Parasitized versus Healthy: *N* = 12; Parasitized versus Control: *N* = 20; Healthy versus Control: *N* = 18). Selective foraging was examined by introducing sunflower seeds into the trials (Parasitized versus Healthy: *N* = 61; Parasitized versus Control: *N* = 13; Healthy versus Control: *N* = 13). Ten sunflower seeds were placed directly in front of the permeable screen at each end of the arena that contained the faeces or control. The faeces and food were separated by a thin screen, and therefore not in direct contact, but the faeces were close enough to the food that direct visual and olfactory investigation was possible (Fig. 1). The amount of time (s) each individual mouse spent in either side of the choice arena and the numbers of sunflower seeds consumed in each of the choice sections were recorded.

### Experiment 2: Nesting Material Choice

This experiment was designed to investigate faecal avoidance in nesting behaviour. Prior to trials in this experiment, used nesting material had to be collected. This was achieved by placing two unused cotton balls in a Sherman live trap overnight during a standard trapping night, and then collecting cotton that had been used for nesting material the following morning. Used cotton balls were easily identified because they had been pulled apart and were large and fluffy, and had urine, faeces, food and seed casings mixed into the cotton. A small subsample of faecal pellets from the cotton was collected and analysed for the presence of intestinal parasites using the same methods described above and labelled ‘healthy’ if the pellets were free from internal parasites, and ‘parasitized’ if they contained at least one species of internal parasite. Samples were then refrigerated until used in the experiment, and for no longer than 36 h.

When Sherman live traps were baited on a subsequent evening, cotton samples from two of the three possible categories, healthy, parasitized and control, were added to the back of each trap, so that a single trap had two different types of cotton balls present. In this experiment 32 wild *Peromyscus* (26 *P. leucopus*; one *P. maniculatus*; five *Peromyscus* where the species was unrecorded) were caught and used. As in experiment 1, the three trials were: (1) used parasitized nesting material versus used healthy material (*N* = 6); (2) used parasitized material versus a control (two unused cotton balls; *N* = 12); and (3) used healthy material versus a control (*N* = 14). The two cotton samples in each trial were randomly dyed red or blue with dilute food colouring, and observers were blind to which treatment the colours signified. After being dyed, the cotton samples were rolled into compact balls matching the size of the unused controls.

Each mouse caught in the trap overnight could choose to use or avoid the two nesting material samples provided. On checking the traps the following morning, we recorded demographic data, as well as whether each of the available samples was used (using the same criteria described above).

### Ethical Note

Traps were set at dusk and checked at dawn. All traps were set with cotton bedding and sufficient crimped oats for a >24 h period. Water was not provided within the traps as *Peromyscus* derive most of their water from their food (MacMillen 1983). All animals were processed within a few hours after checking the traps, so most were released within 8 h of capture. The longest an animal could have been in the trap before being released was no more than 18 h. The metal traps used here protect mice from weather and predation, and in addition, these traps were placed in naturally sheltered areas. Traps from one of the longer-term sampling grids were further protected within metal shelters. We trapped one pregnant, but no lactating, females during the study, but because we did not track individual survival and fitness we were unable to determine whether capture had an effect on her litter. Other studies, with similar methods on this mouse population (Pedersen & Greives 2008), found no significant adverse affects to the population from trapping. Ear tagging is a common and safe method used for wild mice. It does not cause any bleeding, and has no adverse affects on survival for *Peromyscus* spp. (Pedersen 2005). We had IACUC approval for this project through the University of Virginia and the Mountain Lake Biological Station (Protocol No. 3021) and from the Virginia Department of Game and Inland Fisheries (VADGIF No. 022230).

### Analysis

All data were analysed using IBM SPSS v19 (SPSS Inc., Chicago, IL, U.S.A.), unless otherwise stated. To determine whether *Peromyscus* differed from chance in where they spent their time in the choice arena, we used chi-square tests on the counts of which section of the arena each mouse spent the most time. To account for the fact that the centre was longer than either end, individual *G* tests for goodness of fit were used for each mouse to determine where more time, than expected by chance, was spent. Sample sizes within each treatment were small for *P. maniculatus* (mean ± SE = 4.67 ± 1.63), so we report the chi-square results from the more numerous species, *P. leucopus*. The results from both species combined and *P. leucopus* alone are the same, with the exception of the Healthy versus Control treatment without food present, which changes from not significant to significant. Replicated *G* tests for goodness of fit (McDonald 2009) were also used to confirm whether each mouse and, across trials, all mice differed from chance in where they spent their time; however, in some of the trials heterogeneity was significant. The results of both analyses were the same; therefore only the chi-square results are reported. Analyses of the percentage of time spent in the centre section of the choice arena were performed on arcsine-transformed data. The numbers of seeds eaten in each section were analysed using generalized linear models (GZLM) using a negative binomial model with log link function. The use of cotton nesting material was analysed with binary logistic regression with the choice options, cotton dye colour and date in the analyses. All data are presented as mean ± SE.

## Results

### Faecal Avoidance

**Figure 2:**
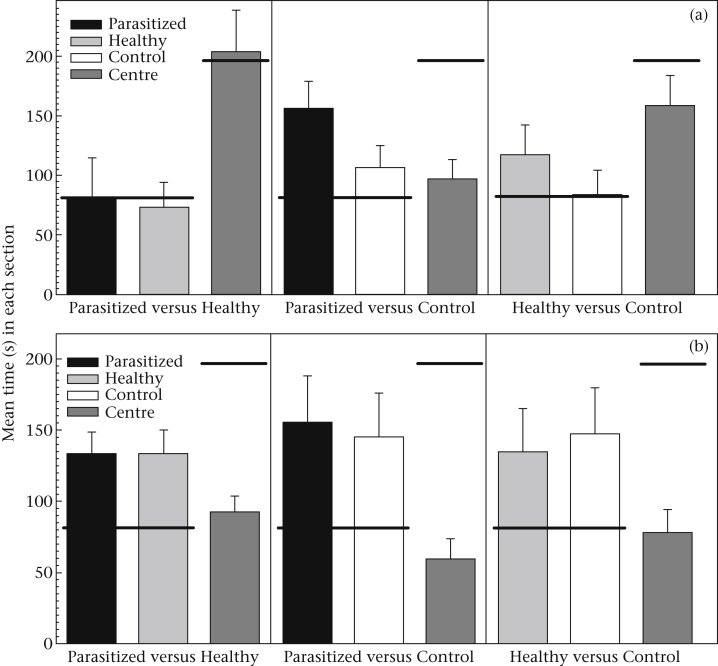
Mean + SE time spent in each section of the choice arena for each of the three treatments, (a) without and (b) with food present. The thick black horizontal bars indicate the expected values for each section.

When no food was provided in the trial, *Peromyscus* differed from chance in where they spent their time in the Parasitized versus Control treatment (chi-square test: ${\text{χ}}_{2}^{2}=7.97$, *P* = 0.02) and the Healthy versus Control treatment (chi-square test: ${\text{χ}}_{2}^{2}=6.47$, *P* = 0.04; Fig. 2a), spending more time near the faecal sample, regardless of infection status, and less time in the centre or near the control than expected. Conversely, in the Parasitized versus Healthy trial, *Peromyscus* did not differ from chance in where they spent their time (chi-square test: ${\text{χ}}_{2}^{2}=0.10$, *P* = 0.61; Fig. 2a). When food was provided and under all three treatment options, *Peromyscus* differed from chance in where they spent their time in the choice arena, always preferring to be near food (chi-square test: Parasitized versus Healthy: ${\text{χ}}_{2}^{2}=50.25$, *P* < 0.0001; Parasitized versus Control: ${\text{χ}}_{2}^{2}=12.97$, *P* < 0.005; Healthy versus Control: ${\text{χ}}_{2}^{2}=11.12$, *P* < 0.005; Fig. 2b).

Mice spent less time in the centre section, and therefore more time in either of the two choice sections, when food was provided than when food was absent (ANOVA: *F*_1,130_ = 19.58, *P* < 0.0001). This was consistent across the three choice treatments (ANOVA: *F*_2,130_ = 0.93, *P* = 0.40). They spent less time in the centre when they were given the choice of parasitized and control treatments, compared to the other two treatments (ANOVA: *F*_2,130_ = 4.41, *P* = 0.01; Fig. 2a, b). The time spent in the centre section did not differ between the sexes (ANOVA: *F*_1,130_ = 0.21, *P* = 0.65) or between species (ANOVA: *F*_1,130_ = 3.47, *P* = 0.07). The gastrointestinal parasite infection status of the focal mouse also did not affect the time it spent in the centre section (ANOVA: *F*_1,130_ = 0.09, *P* = 0.77).

There were no preferences for any of the choices of the faecal infection status available, with mice spending equal time in the parasitized, healthy and control sections, regardless of whether food was present or not (six paired *t* tests: all *P* > 0.27).

### Selective Feeding

**Figure 3:**
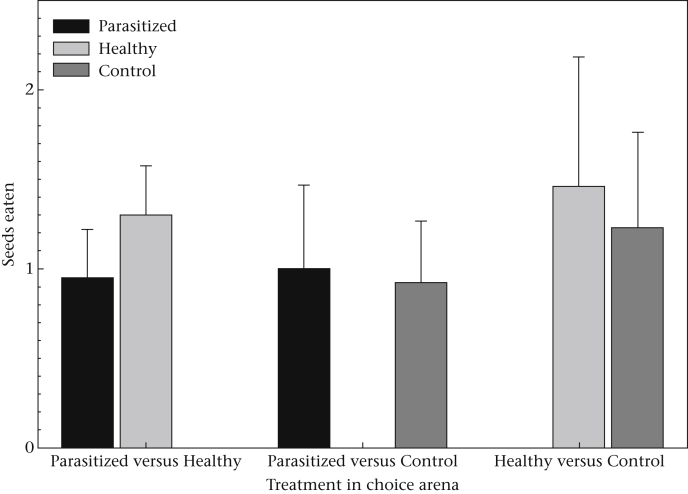
Mean + SE number of seeds eaten in proximity to each of the three choice treatments for the three trials.

Mice were not selective in where they chose to eat. There were no differences in the number of seeds they consumed between the three choice treatments (GZLM: Wald ${\text{χ}}_{2}^{2}=0.58$, *P* = 0.75) or between the presence of parasitized faeces, healthy faeces and no faeces (GZLM: Wald ${\text{χ}}_{2}^{2}=2.15$, *P* = 0.34; Fig. 3). Whether the focal mouse was infected or not did not influence their feeding (GZLM: Wald ${\text{χ}}_{1}^{2}=0.83$, *P* = 0.36), nor did the species differ (GZLM: Wald ${\text{χ}}_{1}^{2}=0.79$, *P* = 0.37). Males consumed 1.38 ± 0.22 seeds, while females only consumed 0.71 ± 0.18 seeds during the trials (GZLM: Wald ${\text{χ}}_{1}^{2}=7.54$, *P* < 0.01).

### Nesting Material Choice

**Figure 4:**
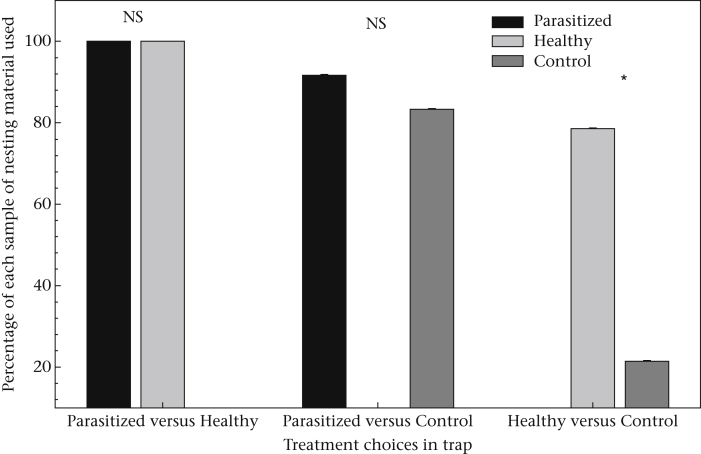
Mean percentage of nesting material samples used in each of the three choice treatments. NS: difference in usage nonsignificant (*P* > 0.54); *P < 0.01.

The colour that the cotton balls were dyed did not influence the nesting material selected by the mice in any of the three choice treatments (binary logistic regression: Parasitized versus Healthy: Wald = 0.00, *P* = 0.99; Parasitized versus Control: Wald = 0.37, *P* = 0.54; Healthy versus Control: Wald = 0.16, *P* = 0.69). When given the choice between used, but healthy, and unused cotton balls, *Peromyscus* preferentially selected the cotton that had been previously used (Wald = 7.16, *P* < 0.01; Fig. 4). However, they showed no preference between parasitized and healthy cotton or parasitized and unused cotton, and in fact usually made a nest including all previously used nesting materials (Fig. 4). The date did not affect whether the nesting material was used (binary logistic regression: Parasitized versus Healthy: Wald = 0.00, *P* = 0.99; Parasitized versus Control: Wald = 0.38, *P* = 0.54; Healthy versus Control: Wald = 2.82, *P* = 0.09).

## Discussion

The results from these experiments suggest that mice do not discriminate between faeces from parasitized and healthy individuals when deciding where to spend time, eat or nest. In fact all the wild mice tested, regardless of their sex, species or gastrointestinal infection status showed no evidence of faecal avoidance or selective foraging, and indeed seemed to prefer being near faeces, regardless of whether the faeces were from parasitized or healthy individuals. These results are surprising and do not support our hypothesis, especially given that the gastrointestinal parasites found in these mice are known to have negative effects on individual fitness (Fuller & Blaustein 1996; Pedersen & Greives 2008).

In the food experiment, mice preferred to spend time near food, regardless of the presence or absence of faeces nearby or the parasite cues from the faeces. Foraging effort is a trade-off between the advantages of feeding (reduced risk of starvation, increased growth or reproduction) and the potential costs (lost opportunity, increased predation risk and parasite transmission; Sih 1987; Lima & Dill 1990). Therefore, in wild *Peromyscus* populations, the advantages of taking feeding opportunities may take precedence over antiparasite behaviours. Furthermore, faeces may actually represent a positive cue for feeding in *Peromyscus*. Larger herbivores aggregate seeds by ingesting fruits from several sources and then defecate the undigested seeds, causing localized concentrations of seeds that may signal a food source (Janzen 1982a, b; LoGiudice 2001; Manzano et al. 2010). While this would not be the case with the faeces of conspecifics, the presence of conspecific faeces may indicate a site where *Peromyscus* have successfully fed. The fact that *Peromyscus* also preferred to spend more time near faeces than away from faeces, even in two of the trials where food was absent, indicates that faeces of conspecifics may be a positive cue beyond just food availability.

This hypothesis is further supported by our findings from the nest material experiments, the results of which are largely consistent with those of the choice arena experiments. Mice preferred to use nesting materials that had been used by other mice in the past, particularly when given the choice between unused cotton and cotton used by a healthy individual. While we acknowledge that our nest choice experiments had small sample sizes, and thus low statistical power to detect differences, we found that in nearly all of the cases, used material, regardless of its infection status, was incorporated into the nests. In both the foraging and nesting experiments, we conclude that faeces acts as a signal to mice that other individuals have been in the same area and possibly gained an advantage by being there, and that this advantage outweighs the risk of parasite infection.

Nesting or sleeping sites, for wild *Peromyscus*, can present a high risk for parasite transmission owing to the timing of oocyst output (Fuller et al. 1995), particularly of *Eimeria*, which is common at our study site (Pedersen 2005). In fact, it is common for other species (e.g. felids and canids) to defecate selectively outside of their nests or dens to reduce the risk of transmission (Hart 1985). Not only did *Peromyscus* in our study readily use most of the nesting material that was provided, but when they did demonstrate a preference it was for used (healthy) over unused material. These results indicate that finding a suitable nest site may outweigh any cost of parasite transmission (Wolff & Hurlbutt 1982; Wolff & Durr 1986) or there may be an actual advantage of selecting nesting material or a nesting site that has been used by conspecifics.

Nests can provide excellent protection from the elements: *Peromyscus* that use nests, and huddle with other mice within a nest, can greatly increase their survival time at low temperatures (Sealander 1952; Glaser & Sheldon 1975). Mice also continue to nest, individually, in the summer (Wolff & Hurlbutt 1982; Madison et al. 1984), indicating that building a nest is important even in the warmer summer months. Old nests or nest material may serve as a cue that the area may be a suitable site, as has been demonstrated in nest site choices in birds (e.g. Eurasian penduline tit, *Remiz pendulinus*: Gergely et al. 2009), where individuals will actively seek to nest on a tree that has an old nest on it. Additionally, or alternatively, the use of old materials may simply reflect the greater costs associated with looking for new material, not nesting at all or building a new nesting site (Jackson & Tate 1974; Blem et al. 1999). In all of these animals, the risks posed by parasite infection may be less significant than the cost of building a new nest at a new site.

Ultimately, our study shows that wild *Peromyscus* exhibit different behaviour, with respect to faeces, than ungulates and domesticated animals, which selectively feed or defecate to reduce opportunities for parasite transmission (Hart 1994). A variety of mechanisms could explain the absence of selective feeding and faecal avoidance while foraging or nesting in wild *Peromyscus*. First, domestic and laboratory animals are likely to be exposed to significantly fewer pathogens or treated to remove infection (Abolins et al. 2011; Boysen et al. 2011), so their response to the potential for transmission may be intensified. This contrasts with wild *Peromyscus*, which encounter pathogens on a daily basis and are more accustomed to their presence, or may already be infected when they encounter parasitized faeces. In our study population more than 76% of mice are infected with gastrointestinal parasites, and more than 51% are co-infected with two or more parasite species (Pedersen 2005). Second, domesticated grazing animals and laboratory mice may have the option of practising selective feeding because they live in largely controlled environments, where food is abundant (LoGiudice & Ostfeld 2002). Finally, predation risk among livestock and laboratory animals is minimal, allowing individuals to express greater selectivity in what they will accept to eat as well as the risks that they can take to forage or nest. However, while several studies have demonstrated faecal avoidance and selective foraging in ungulates (e.g. Ezenwa 2004) and laboratory mice (e.g. Kavaliers et al. 1997), other studies in bushbucks, *Tragelaphus scriptus* (Apio et al. 2006) have not found evidence for other potential antiparasite behaviours, specifically localized defecation sites. Food availability varies across species, time frames and geographical regions, and may be an important factor determining whether parasites, predation risk, reproduction or food limitation exerts the most pressure on each of these species (Abrams 1991).

Understanding antiparasite behaviours in wild animals is an important step in gaining insight into the response of individuals to the risk of infection, and the consequences that follow for host–parasite dynamics and parasite transmission. The results of these experiments suggest that there is a trade-off between the risks of avoiding parasites and the demands of living in the wild, and that in wild *Peromyscus*, immediate selection pressures such as food shortage and predation may influence behaviour more than parasites do.
